# Influence of Gait Speed on Inter-Joint Coordination in People with and Without Parkinson’s Disease

**DOI:** 10.3390/bios15060367

**Published:** 2025-06-06

**Authors:** Patrick Ippersiel, Philippe C. Dixon, Elke Warmerdam, Robbin Romijnders, Walter Maetzler, Clint Hansen

**Affiliations:** 1 Centre de Recherche Azrieli du CHU Sainte-Justine, Montreal, QC H3T 1C5, Canada; patrick.ippersiel@umontreal.ca; 2 School of Kinesiology and Physical Activity Sciences, Faculty of Medicine, University of Montreal, Montreal, QC H3T 1J4, Canada; 3 Department of Kinesiology and Physical Activity, McGill University, 475 Av des Pins O 2nd Floor, Montreal, QC H2W 1S4, Canada; phil.dixon@mcgill.ca; 4 Biomedical Signals and Systems, Faculty of Electrical Engineering, Mathematics and Computer Science, University of Twente, 7522 Enschede, The Netherlands; e.warmerdam@utwente.nl; 5 Department of Neurology, University Hospital Schleswig-Holstein Campus Kiel, Kiel University, Arnold-Heller Str. 3, 24105 Kiel, Germany; r.romijnders@neurologie.uni-kiel.de (R.R.); w.maetzler@neurologie.uni-kiel.de (W.M.)

**Keywords:** inertial measurement units, gait analysis, Parkinson’s disease, coordination, coordination variability

## Abstract

Background: The influence of gait speed on lower-extremity coordination while walking in people with Parkinson’s disease (pwPD) is poorly understood. This study sought to investigate the relationship between gait speed and hip–knee coordination and coordination variability in older adults and pwPD. Methods: A total of 27 pwPD and 21 healthy older adults were recruited. Participants walked in a straight line at slow, preferred, and fast walking speeds. Gait data were collected using inertial measurement units, and the kinematics of the hip and knee were calculated. Coordination and coordination variability at the hip–knee joint pair were determined using continuous relative phase. A repeated measures two-way ANCOVA tested the impact of gait speed on coordination and coordination variability, while group differences were evaluated using statistical parametric mapping (SPM). Results: Neither the healthy older adults nor the pwPD adjusted their hip–knee coordination in response to changes in gait speed. pwPD also displayed a trend towards restricted hip and knee joint excursion compared to older adults, which may further limit their ability to adapt gait strategies. Conclusions: These findings suggest that interventions addressing both joint excursion and motor adaptability may be important for improving gait function in individuals with Parkinson’s disease. Real-world applicability can be found in the potential of wearable sensors to become a valuable tool in routine clinical practice for both diagnosis and ongoing management. Trial registration: The study is registered in the German Clinical Trials Register (DRKS00022998).

## 1. Introduction

Parkinson’s disease (PD) is an age-related, progressive neurodegenerative disorder characterized by the disruption of the motor system [1]. This translates to difficulties with movement planning and sequencing, stemming from symptoms such as bradykinesia, postural instability, and rigidity [2]. In people with PD (pwPD), these symptoms negatively impact their ability to walk, resulting in reduced gait speed and an increased risk of falling [3,4]—factors which can impact their quality of life and social participation [5,6]. Specifically, reductions in gait speed with aging have also shown predictive capacity for adverse health outcomes, and they may be an important clinical marker [7]. Recently, von der Recke et al. [8] showed that pwPD have a reduced range of gait speed compared to healthy older adults (HOAs), driven by step time in the low and step length in the high gait speed range. This is particularly important when considering environmental factors, as people tend to walk slower in unsupervised settings (e.g., home environment [9]) and utilize a broader range of gait speed (RGS) in daily life [10] compared to clinical or supervised assessments.

At lower gait speeds, stride length and swing time are affected [11]. While these metrics underscore modifiable gait characteristics, these variables provide limited insight into neuromuscular control [12], especially in the context of a reduced RGS. Thus, clinical interventions simply focused on increasing gait speed may neglect other parameters which could improve function and the quality of life for pwPD. Kinematic features such as inter-joint coordination and its variability describe neuromuscular control and may be particularly interesting for (non-pharmacological) treatment approaches [13]. Inter-joint coordination describes coupling and timing relationships between adjacent joints during movement (e.g., hip–knee coupling during gait). Coordination variability quantifies the within-individual variability in joint coupling during movement [14]. In clinical populations, excessive coordination variability during gait is suggestive of unstable neuromuscular control, while reduced coordination variability points to an overly rigid motor system [15]. Past work identified a less variable and more tightly coupled gait strategy in pwPD, compared to HOAs [16], suggesting “coordination rigidity” as a possible feature of PD.

The use of wearable sensors such as inertial measurement units (IMUs) allows for the quantification of gait kinematics (e.g., joint angles) and physical measures like gait speed [17,18], providing insight into adverse outcomes in HOAs, including falls [19]. The relationship between gait speed and coordination in PD is unclear, but pwPD show a reduced ability to adapt their gait strategy in response to changing speeds compared to HOAs [20]. Hence, this study sought to investigate the relationship between gait speed and lower-extremity coordination and coordination variability in HOAs and pwPD. By examining gait speed and related parameters, we aimed to explore how these advanced metrics can be used in clinical practice. We hypothesized that pwPD would be less adaptable to changes in gait speed and that more tightly coupled and variable lower-extremity coordination would be observed in pwPD [16].

## 2. Methods

### 2.1. Study Overview and Ethics

This cross-sectional study describes one aspect of a larger study registered in the German Clinical Trials Register (DRKS00022998, registered on 4 September 2020). Study procedures were approved by the ethics committee of the Medical Faculty of Kiel University (D438/18) and conducted in accordance with the principles of the Declaration of Helsinki. All study participants provided informed consent. Please refer to [21] for the full assessment procedures.

### 2.2. Participants

A total of 47 older adults with (pwPD n = 27) and without PD (HOAs n = 21) participated in this study, where PD was defined according to the Movement Disorder Society (MDS) criteria [22]. All pwPD completed the study procedures 30–120 min after the administration of dopaminergic medication (Levodopa). The pwPD were able to walk without a walking aid, and based on the MDS-UPDRS III scores of 30 ± 21 and Hoehn–Yahr scores of 2.5 ± 1, they were considered mild to moderately affected.

Participants were included if they were aged >60 years and able to walk independently without an aid. Participants were excluded if they were judged to have a mobility disorder impacting walking performance or scored <15 on the Montreal Cognitive Assessment tool.

### 2.3. Data Collection

Participants were equipped with 15 IMUs (Noraxon USA Inc., myoMOTION, Scottsdale, AZ, USA), containing a triaxial accelerometer (+/−16 g), gyroscope (+/−2000 °/s), and magnetometer (+/−1.9 Gauss). IMUs were attached to the head, sternum, upper arms, forearms, pelvis, thighs, medial shanks (proximal), lateral ankles, and the dorsum of the feet and secured with an elastic band (Supplementary Figure S1). Data were sampled at 200 Hz. For this analysis, IMU data from the pelvis, right thigh, and right shank were retained.

### 2.4. Walking Protocol

Participants walked on a 5 m long and 1 m wide walkway. They were instructed to walk at three different speeds: slow, preferred, and fast. To ensure steady gait measurement and to limit variations related to gait initiation/termination [8], participants started walking 2 m before the 5 m track and ceased walking 2 m after the track.

### 2.5. Data Processing

Raw IMU data were imported into MATLAB (Matlab R2024b, Version 24.2.0.2863752; The MathWorks Inc., Natick, MA, USA) for processing with biomechZoo [23] and custom code (see: https://github.com/PatIpps/Parkinson-CRP- (accessed on 9 October 2024)). Acceleration, gyroscope, and magnetometer data were converted into m/s^2^, rad/s, and uT, respectively. IMU data were then filtered using a 4th-order low-pass Butterworth filter, with a cut-off frequency of 6 Hz.

### 2.6. Joint Angle Extraction and Event Detection

Sagittal plane hip and knee joint angles were determined based on the Xsens MVN algorithm [24]. Sensor quaternion orientations were calculated via the fusion of accelerometer, gyroscope, and magnetometer signals, using the complementary filter function in the Sensor Fusion and Tracking Toolbox in MATLAB [25]. To estimate Euler angles, the conjugate of the proximal sensor quaternions was multiplied by the distal sensor quaternions for each time stamp. The resulting quaternion orientations for the hip and knee were converted to Euler angles in the Z (medial–lateral), Y (vertical), X (anterior–posterior) axis rotation sequence [24,26], and sagittal plane hip and knee joint angles were retained. Gait events (initial contact) were identified using the first zero-crossing after the mid-swing peak in the sagittal plane angular velocity of the shank sensor [27]. For each participant and walking speed, hip and knee joint angles and gait events were visually inspected. If judged to be erroneous, gait events were manually adjusted. Manual adjustments were made infrequently, occurring in less than 5% of the total analyses.

### 2.7. Joint Excursion Calculation

The sagittal plane joint excursions of the hip and knee were calculated using all available cycles for each participant. For each gait cycle, the minimum value was subtracted from the maximum value and then averaged, separately, for the three walking speeds.

### 2.8. Coordination and Coordination Variability Calculations

Inter-joint coordination was calculated using continuous relative phase (CRP) analysis [28,29]. CRP analysis provides an instantaneous measure of coordination between two adjacent joints and describes how coupled (i.e., in-phase, moving synchronously), or uncoupled (i.e., anti-phase, moving asynchronously), a joint pair behaves. Analyses were performed as per Lamb and Stökl and were described in [29]. Briefly, extraneous kinematic data were retained on both ends of the signal. Phase angles for the hip and knee were determined using a Hilbert transform. The absolute difference between the hip and knee phase angles quantified the CRP angle for the hip–knee joint pair. The resulting values were restricted to a [0, 180] scale, where values closer to 0 indicate in-phase behavior (i.e., joints moving in same direction), while values closer to 180 indicate anti-phase behavior (joints moving in opposite direction). Finally, extraneous data were removed. In sum, hip–knee CRP angles were calculated for the entire waveform, for each participant, for each walking speed. CRP data were segmented into individual gait trials and time-normalized to 101 data points.

To determine a summative metric for coordination and coordination variability, the Mean Absolute Relative Phase (MARP) and Deviation Phase (DP) were used, respectively [15,30]. The MARP refers to the average coordination strategy between the hip and knee during gait (i.e., mean in-phase/anti-phase hip–knee coupling relationship). The MARP was determined by taking the mean of the ensemble averaged CRP waveforms, at the participant level. The DP refers to the within-individual between-trial variability in the hip–knee coupling relationship across gait cycles (i.e., how variable their hip–knee coordination strategy is). The DP was determined by averaging the standard deviations of individual participant ensemble average curves, over the gait cycle. The MARP and DP were calculated for the hip–knee joint pair, for each participant, across each walking speed.

### 2.9. Statistical Analysis

Demographic and clinical variables were explored and tested for normality using the Shapiro–Wilk test. Descriptive statistics were calculated for demographic and clinical variables and presented as the mean (standard deviation). Group comparisons for demographic and clinical variables were made using independent sample t-tests or Mann–Whitney U tests. Statistical significance was set at α = 0.05. Effect sizes were reported using Cohen’s d.

A two-way analysis of variance (ANOVA) was conducted to examine the effects of group (pwPD and HO) and walking condition (slow, preferred, fast) on walking speed. The results of the ANOVA revealed a significant main effect of group (F(1, 120) = 8.83, *p* < 0.01). This indicates that there was a significant difference in walking speed between the two groups, regardless of walking conditions.

There was also a significant main effect of walking condition (F(2, 120) = 4.53, *p* < 0.001). This demonstrates that walking speed significantly varied across the three conditions.

The interaction effect between group and walking condition was not significant (F(2, 120) = 0.16; *p* = 0.06). This suggests that the effects of the walking condition did not differ depending on the group (HO vs. pwPD).

To further explore the significant main effects, Bonferroni post hoc tests were conducted. Post hoc comparisons revealed that HO had a significantly higher average walking speed (mean = 1.06; SD = 0.39) compared to the pwPD (mean = 0.94; SD = 0.33; *p* < 0.01). Across both groups, post hoc analyses showed the following significant differences in walking speed.

Walking speed in the fast condition (mean = 1.31; SD = 0.33) was significantly higher than that in the preferred (mean = 1.02; SD = 0.20; *p* < 0.001) and slow (mean = 0.66; SD = 0.17; *p* < 0.001) conditions. Walking speed in the preferred condition was significantly higher than that in the slow condition (mean = 0.66; SD = 0.17; *p* < 0.001).

Hence, to test the hypothesis that gait speed influences hip–knee joint excursion, coordination, and variability, ANCOVAs were run for gait speed (slow, preferred, fast) and group (pwPD and HOAs), with the actual walking speed as a covariate. Data distribution was assessed for normality and homogeneity of variance using the Shapiro–Wilk and Mauchly’s tests, respectively. In the event of a significant main effect, Bonferroni-adjusted post hoc paired t-tests or Wilcoxon Signed-rank tests were performed (α = 0.05). Effect sizes were reported using partial eta^2^ and Kendall’s W for the ANCOVA and Friedman tests, respectively.

To test the hypothesis that pwPD would have a more in-phase (smaller MARP) and less variable (lower DP) hip–knee coordination strategy, statistical parametric mapping (SPM) was used [31]. Two-tailed independent sample t-tests (spm1d.stats.ttest2) or the non-parametric equivalent (spm1d.stats.nonparam.ttest2) compared the pre-processed time series data of the entire gait cycle for the MARP and DP, in pwPD and HOAs, at three different speeds (slow, preferred, fast). Statistical significance was set at α = 0.05.

SPM analyses were implemented using the open-source spm1d code (v.M0.1, www.spm1d.org) in MATLAB, while other analyses were performed in SPSS (IBM Corp, 2023, Version 29.0.1.0, Armonk, NY, USA).

## 3. Results

**Figure 1:**
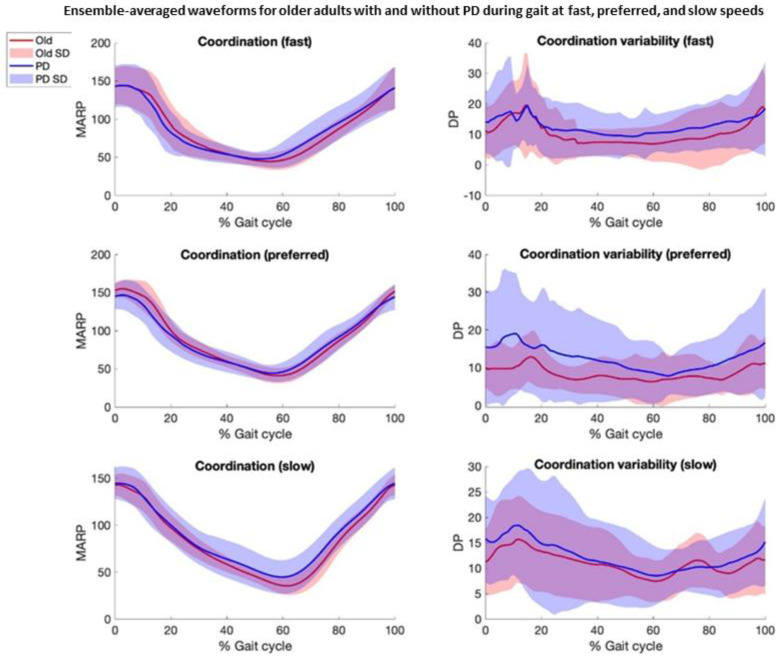
Ensemble-averaged waveforms +/− 1 standard deviation (SD) for older adults with and without Parkinson’s disease (PD) during gait at fast, preferred, and slow speeds. Waveforms depict hip–knee coordination (Mean Absolute Relative Phase, MARP) and hip–knee coordination variability (Deviation Phase, DP). Red line represents older adults, and blue line represents adults with PD. Shaded areas represent SD.

Based on visual inspection, data from two older adults and three participants with PD were identified as possible outliers. These data were subsequently confirmed and excluded based on their values exceeding the threshold of a greater or less than 1.5 × interquartile range. Therefore, data from 19 older adults and 24 participants with PD were retained for analyses. Demographic, clinical variables, walking speeds, and the average number of steps are listed in Table 1. Ensemble-averaged waveforms for the coordination and coordination variability parameters are presented in Figure 1.

### 3.1. Results of ANCOVA for Impact of Gait Speed on Hip–Knee Coordination (MARP) and Hip–Knee Coordination Variability (DP), by Group

Two-way ANCOVAs were performed to examine the effects of gait speed and group on the DP, MARP, and knee and hip joint excursions, after controlling for walking speed.

There were no statistically significant two-way interactions between gait speed and group for the MARP (F(2, 116) = 0.95; *p* = 0.39) or DP (F(2, 116) = 0.93; *p* = 0.40) whilst controlling for actual walking speed.

Therefore, no analyses of simple main effects for groups or gait speed were performed.

### 3.2. Analyses for Hip–Knee Coordination (MARP) and Hip–Knee Coordination Variability (DP)

**Figure 2:**
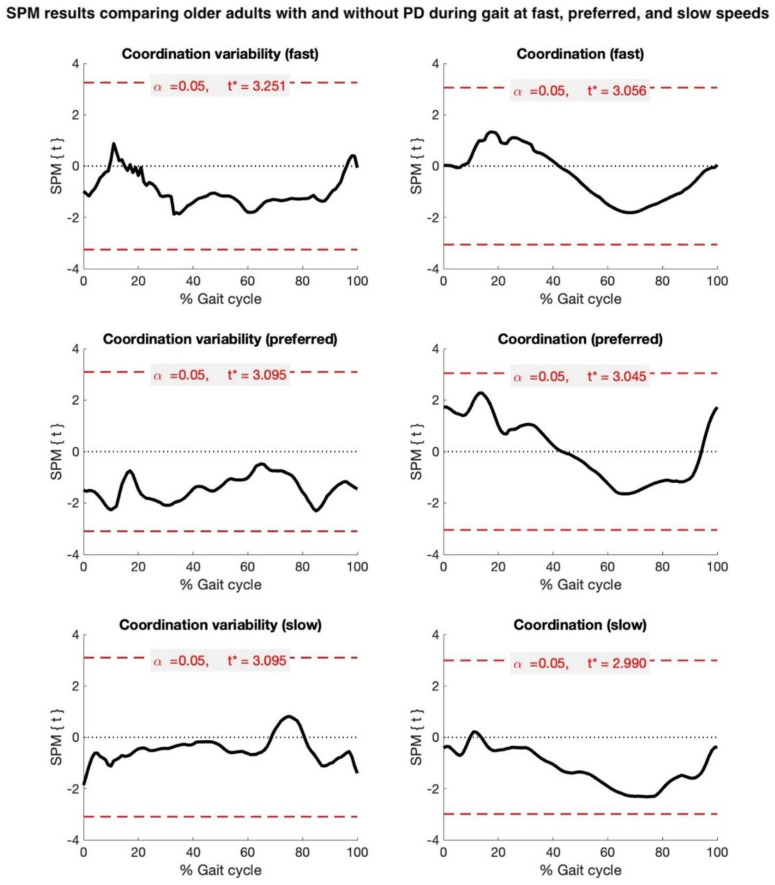
Statistical parametric mapping (SPM) results comparing older adults with and without Parkinson’s disease (PD) during gait at fast, preferred, and slow speeds. Black waveforms depict extent of group difference in hip–knee coordination (Mean Absolute Relative Phase, MARP) and hip–knee coordination variability (Deviation Phase, DP), referenced to healthy older adults. Dashed red line denotes critical t-threshold (t*) for statistical significance.

Based on the SPM analyses of temporally aligned gait data, the metrics of hip–knee coordination and coordination variability were not statistically different between groups. For coordination, there was a trend toward the significance of more in-phase coordination (tightly coupled, smaller MARP) in pwPD, compared to HOAs, during the stance phase (preferred and fast speeds), and more anti-phase coordination (loosely coupled, greater MARP) during the swing phase at the slow, preferred, and fast speeds. For coordination variability, there was a trend toward the significance of a more variable gait strategy (greater DP) in pwPD across most of the gait cycle, predominantly at the preferred and fast speeds (Figure 2).

### 3.3. Results of ANCOVA for Impact of Gait Speed on Hip and Knee Joint Excursion, by Group

Compared to HOAs, pwPD walked with a smaller hip and knee joint excursion at all speeds (Table 2); however, this was not statistically significant. The two-way ANCOVAs did not show any statistically significant two-way interactions between gait speed and group for knee joint excursion (F(2, 116) = 0.53; *p* = 0.59) or hip joint excursion (F(2, 116) = 0.09; *p* = 0.91) whilst controlling for actual walking speed.

Therefore, no analyses of simple main effects for groups or gait speed were performed.

## 4. Discussion

This study investigated the impact of gait speed on lower-extremity coordination and coordination variability in pwPD and HOAs, focusing on hip–knee couplings and joint excursion. We hypothesized that pwPD would be less adaptable to changes in gait speed and that more tightly coupled (in-phase) and variable lower-extremity coordination would be observed in the PD group.

### 4.1. Gait Speed Does Not Significantly Influence Coordination Variability, Independent of Clinical Status

Speed had no significant effects on coordination and coordination variability for HOAs and pwPD. Compared to HOAs, pwPD walked with a smaller knee joint excursion at all speeds (Table 2); however, this was not statistically significant.

Previous research showed that reducing gait speed led to more in-phase (tightly coupled) hip–knee coordination and that slower walking demands increased dynamic stability [32], which could translate to more rigid, in-phase hip and knee coupling to maintain balance and stability. Healthy adults can flexibly adjust their coordination strategy based on speed, adopting more coupled movement patterns at slower speeds to optimize gait mechanics, which are affected while walking, particularly in pwPD [20]. This lack of adaptability is consistent with the motor rigidity and bradykinesia commonly observed in PD, where the neurodegenerative process affecting the basal ganglia limits the capacity to modify motor control in response to task demands [2]. As the results do not show significant group differences in coordination variability, it can be concluded that gait speed had no impact on variability in either group. For both HOAs and pwPD, the degree of hip–knee coordination variability remained stable across the slow, preferred, and fast speeds. This result contrasts with our hypothesis, which anticipated greater variability in the PD group due to impaired motor control reflecting subtle motor instability in PD that may still be clinically relevant. For instance, previous research suggests that greater gait variability in PD may reflect unstable or impaired motor control [15,33], a behavior which might be accentuated under more demanding conditions like faster walking speeds.

The time series analysis of hip–knee coordination across the gait cycle did not reveal any significant group differences, but there were trends toward the significance of different coordination strategies between the two groups. Specifically, pwPD displayed more in-phase (tightly coupled) coordination during the stance phase at the preferred and fast speeds compared to HOAs. In contrast, they exhibited less in-phase (more loosely coupled) coordination during the swing phase across all speeds. These trends suggest that while group differences in coordination were not statistically significant, individuals with PD may rely more on tightly coupled movements during the weight-bearing stance phase, likely due to their impaired balance and motor control, which forces them to adopt more rigid coordination strategies. During the swing phase, however, they may exhibit less coupled movement patterns as a compensatory strategy to accommodate for deficits in dynamic control.

### 4.2. Joint Excursion During Gait Does Not Differ Between HOAs and pwPD

Differences in hip and knee joint excursion were observed between the two groups; however, the two-way interaction remained non-significant. Compared to OAs, pwPD had reduced hip joint excursion at all walking speeds. More notably, pwPD consistently displayed smaller knee joint excursion across all walking speeds. While these findings are not supported statistically, they do align with previous research showing that PD is associated with reduced joint excursion, which may reflect motor rigidity and bradykinesia in pwPD [34]. Restricted joint excursion, particularly in the knee, could contribute to the overall impairment in gait mechanics seen in PD, further limiting the ability of pwPD to adapt their gait to varying speeds.

The reduced joint excursion observed, particularly in the knee joint, may also explain the absence of significant speed-dependent changes in coordination in pwPD. With a constrained movement range, pwPD likely have fewer degrees of freedom to adjust their coordination strategy, resulting in a more rigid and less adaptable gait pattern. Thus, interventions aiming to improve joint excursion could be beneficial for enhancing the gait adaptability of pwPD.

### 4.3. Clinical Implications

Our study findings have important clinical implications for the treatment and rehabilitation of pwPD. While the reduced adaptability of coordination in response to gait speed changes was not shown during the short walking episodes, the limited joint excursion might suggest that therapeutic interventions should focus on enhancing movement flexibility and adaptability. Gait training that incorporates varying speeds, alongside exercises targeting joint flexibility (especially at the hip and knee), may help improve the motor adaptability of pwPD. Additionally, interventions focusing on increasing joint excursion and reducing motor rigidity could mitigate some of the observed deficits in lower-extremity coordination.

### 4.4. Limitations and Future Directions

Several limitations should be noted. First, the relatively small sample size may have limited the ability to detect subtle group differences in coordination variability and coordination strategies. Future research with larger, more diverse samples could provide more definitive conclusions. Additionally, while we focused on hip–knee coordination, examining other aspects of motor control, such as muscle activation patterns or the coordination of other lower-extremity joints, could offer a more comprehensive understanding of gait impairments in PD. Lastly, future studies should investigate the potential role of disease severity in gait coordination and variability, as our sample included individuals with mild to moderate PD, and different results may be observed in individuals with more advanced disease.

## 5. Conclusions

Neither HOAs nor pwPD adjust their hip–knee coordination in response to changes in gait speed. No significant group differences were found in coordination variability, though a trend toward greater variability in pwPD at faster speeds was observed. pwPD also displayed a trend towards restricted hip and knee joint excursion compared to HOAs, which may further limit their ability to adapt gait strategies. These findings highlight the need for interventions targeting both joint excursion and motor adaptability to improve gait function in pwPD.

## Figures and Tables

**Table 1:** Descriptive parameters of demographic and clinical variables (Montreal Cognitive Assessment (MoCA) and Short Physical Performance Battery (SPPB)) in healthy older adults (HOAs) and people with Parkinson’s disease (pwPD).

	Age [Years]	Height [cm]	Weight [cm]	MOCA	SPPB	Slow [m/s] (# of Steps)	Preferred [m/s] (# of Steps)	Fast [m/s] (# of Steps)
PD n = 24 10 ♀, 16 ♂	65 ± 9	174 ± 9	81 ± 16	24 ± 3	9.1 ± 2.1	0.65 ± 0.17 5 ± 2	0.97 ± 0.18 3 ± 1	1.19 ± 0.34 3 ± 1
OA n = 19 11 ♀, 8 ♂	71 ± 7	173 ± 9	77 ± 17	25 ± 4	10.4 ± 1.7	0.67 ± 0.17 5 ± 1	1.07 ± 0.21 3 ± 1	1.45 ± 0.27 3 ± 1

♀ = male sex, ♂ = female sex.

**Table 2:** Descriptive parameters comparing hip and knee joint excursions during walking at slow, preferred, and fast speeds in older adults (HOAs) and people with Parkinson’s disease (pwPD).

Gait Speed	Hip Joint Excursion		Knee Joint Excursion	
	Older Adults	pwPD	Older Adults	pwPD
Slow	34.6 (7.4)	30.2 (7.4)	59.6 (8.1)	53.4 (8.4)
Preferred	40.5 (7.6)	34.5 (7.3)	65.2 (8.6)	55.3 (9.2)
Fast	45.2 (7.6)	39.5 (10.5)	62.6 (9.7)	55.5 (9.7)

Mean and standard deviation values are presented.

## Data Availability

The data presented in this study are available on request from the corresponding author due to privacy reasons.

## Supplementary Materials

The following supporting information can be downloaded at: https://www.mdpi.com/article/10.3390/bios15060367/s1, Figure S1. (a) shows the placement of the 15 IMUs on the body for the walking trials. (b) shows the experimental setup of the 5m walking path on the walkway in the clinical gait laboratory for the forward walking trials.
