# Supplementary material for: Influence of Gait Speed on Inter-Joint Coordination in People with and Without Parkinson’s Disease
Source: Biosensors (Basel). 2025 Jun 6;15(6):367. doi: 10.3390/bios15060367 (PMC12190309; doi:10.3390/bios15060367)
Supplement: Supplementary file 1 [file biosensors-15-00367-s001.zip › biosensors-3566232-supplementary.pdf]

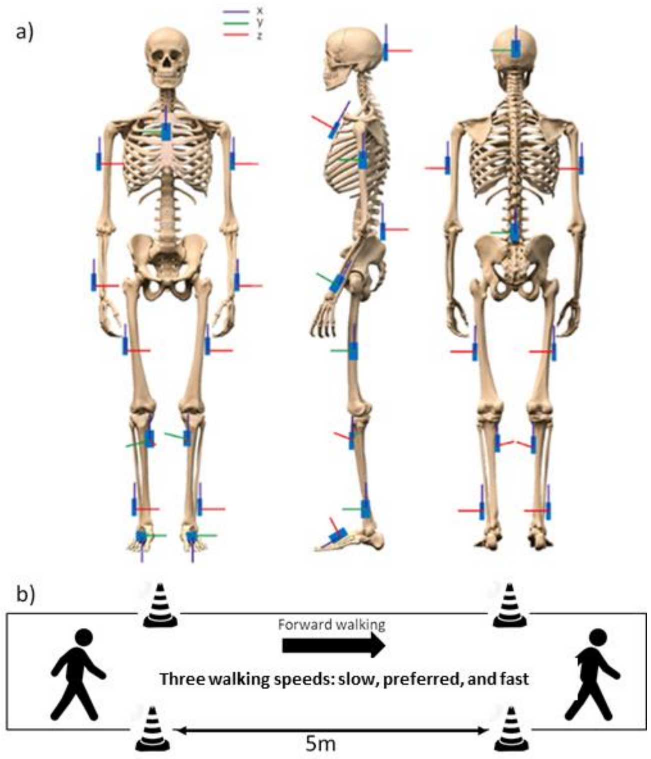

Figure S1. a) shows the placement of the 15 IMUs on the body for the walking trials. (**b**) shows the experimental setup of the 5m walking path on the walkway in the clinical gait laboratory for the forward walking trials.
